# Amiodarone: A Newly Discovered Association with Bilateral Vestibulopathy

**DOI:** 10.3389/fneur.2018.00119

**Published:** 2018-03-06

**Authors:** Robert Gürkov

**Affiliations:** ^1^Department of Otorhinolaryngology, Ludwig-Maximilians-Universität München, Munich, Germany

**Keywords:** head impulse test, inner ear, vertigo, ototoxicity, adverse drug reactions

## Abstract

**Background:**

Bilateral vestibulopathy (BVP) is a debilitating disorder characterized by the hypofunction of both vestibular end organs or nerves. The most frequent identifiable causes of BVP are ototoxic drug effects, infectious and autoimmune disorders. However, the majority of cases remain idiopathic. Very recently, the first discovery of a clinical case of Amiodarone-associated BVP has been reported.

**Methods:**

An overview of the literature concerning the relation between amiodarone toxicity and BVP is presented and discussed.

**Results:**

Older reports on amiodarone-induced symptoms of vertigo and gait instability lack a description of vestibular function test results. Recent evidence from retrospective studies including vestibular function testing in patients taking amiodarone have identified the drug as the hitherto unsuspected potential cause of a relatively large proportion of cases with “idiopathic” BVP.

**Conclusion:**

Patients who receive amiodarone should be monitored with vestibular function testing in order to recognize potential adverse effects on the vestibular system and allow for an informed decision on possible drug reduction or withdrawal.

Bilateral vestibulopathy is a debilitating disorder characterized by the hypofunction of both vestibular end organs or nerves. It accounts for about 3% of the diagnosis in a tertiary neurotology clinic (1), and its prevalence has been reported as high as 81 per 100,000 (2). The most frequent identifiable causes of BVP are ototoxic drug effects, infectious and autoimmune disorders (3). The majority of cases, however, remain idiopathic. Very recently, the first discovery of a clinical case of amiodarone-associated BVP was reported (4). Here, the existing evidence for the concept of amiodarone-induced BVP is summarized and discussed.

Amiodarone is an iodinated benzofuran derivative (Figure 1) with Class I, II, III, and IV antiarrhythmic properties. It is the most commonly used antiarrhythmic drug for the indication of supraventricular and ventricular arrhythmias. It has a long elimination half-life of about 50–140 days, and therefore, it may take several months before an adverse effect is reversed when the drug is stopped (5, 6).

**Figure 1:**
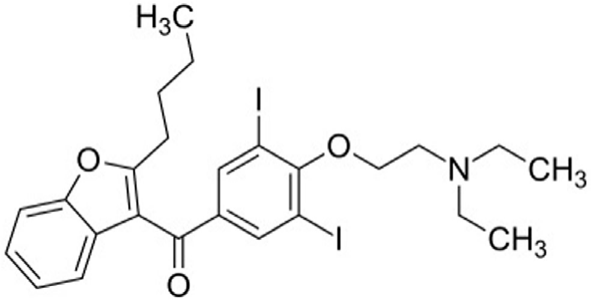
Structural formula of amiodarone.

Thorough follow-up is essential to the care of patients taking amiodarone. Adverse effects are common, with prevalence rates reaching 15% during the first year of use and 50% during long-term use (Table 1). Especially when amiodarone is used for non-life-threatening arrhythmias, such as atrial fibrillation, the risk may outweigh the benefit if serious adverse effects occur. In a recent study in patients below 65 years of age with atrial fibrillation, the drug was discontinued in the first year by 52% (7).

**Table 1:** Overview of amiodarone-induced adverse effects [modified after Ref. (8)].

System	Adverse effect	Incidence (%)
Neurologic	Ataxia, paresthesia, neuropathy, sleep disturbance, memory disturbance, tremor	3–30

Ocular	Halo vision	<5
	Optic neuropathy	<1
	Photophobia, visual blurring, microdeposits	>90

Thyroid	Hypothyroidism	4–22
	Hyperthyroidism	2–12

Pulmonary	Cough, dyspnea	2

Cardiac	Bradycardia, AV block	5
	Ventricular proarrhythmia	<1

Gastrointestinal	Nausea, anorexia, constipation	30
	AST/ALT level increase	15–30
	Hepatitis, cirrhosis	<3

Genitourinary	Epididymitis, erectile dysfunction	<1

Cutaneous	Blue discoloration	<10
	Photosensitivity	25–75

*AST, aspartate aminotransferase; ALT, alanine aminotransferase; AV, atrial-ventricular*.

## Adverse Effects of Amiodarone

The reported frequencies of neurologic adverse effects are quite variable, ranging from 2.8 (9) to 28% (10). Also, the correlation between therapy duration and adverse effect severity is not uniformly reported, but was found to be positive in one study (9). Neurological complications are not always reversible with amiodarone discontinuation (11). High doses have even been reported to cause quadriplegia. Muscle weakness and tremor are more frequent findings. The neuropathy seems to be rather of the demyelinating type than the axonal loss type. The management strategy for neurological toxicity is to discontinue or decrease the amiodarone dose and wait for its elimination or decreased effects. Concerning the dose–adverse effect relationship, Table 2 shows the impressive correlation between the daily amiodarone maintenance dose and the frequency of neurotoxic adverse effects in previously published reports.

**Table 2:** Prevalence of amiodarone neurotoxicity according to the daily maintenance dose.

Source	Mean or usual daily dose (mg)	Prevalence of neurotoxic effects (%)
Greene et al. (12)	600	74
Morady et al. (13)	600	35
Charness et al. (14)	580	54
Palakurthy et al. (15)	468	44
Coulter et al. (10)	277	27.5
Vorperian et al. (16)	152–330	4.6
Cairns et al. (17)	208–308	3.1
Orr and Ahlskog (9)	223.8	2.8
Ahmed et al. (18)	200	1.6
Julian et al. (19)	200	0.5

*In previously published series, there is an impressive correlation between the daily amiodarone maintenance dose and the frequency of neurotoxic adverse effects [modified after Ref. (9)]*.

Toxic effects on the thyroid may cause both hypo- and hyperthyroidism. Typically, amiodarone-induced hypothyroidism occurs within the first 1–24 months of treatment (20). Amiodarone-induced thyrotoxicosis is less prevalent. It can occur suddenly at any time during or even months after treatment. Since amiodarone also exerts beta-blocking effects, the typical signs of thyrotoxicosis are often missing. Common findings include loss of weight or a significant change in warfarin dosing (8).

Pulmonary amiodarone-induced toxicity most commonly presents as diffuse interstitial lung disease or hypersensitivity syndrome that may mimic an infection. It generally presents in the first year of therapy with acute or subacute cough; later symptoms include progressive dyspnea and, occasionally, fever. Other manifestations include respiratory distress syndrome, pulmonary nodules or solitary masses, or pleural effusions (8).

In the gastrointestinal system, nausea, anorexia, and constipation are relatively common side effects of amiodarone. They are dose-related and usually do not require specific interventions. In less than 1% of the patients treated annually, clinically significant liver toxicity occurs.^1^ Liver dysfunction is more frequent when high doses are used and with a long treatment duration. Given the potential accumulation and persistence of amiodarone in hepatic tissue, even a long time after the cessation of therapy, the total cumulative dosage may play a prominent role. Typical symptoms include nausea, loss of weight, fatigue without jaundice, and the examination reveals an enlarged liver and elevated serum aminotransferase and alkaline phosphatase levels.

## Indirect Evidence for Amiodarone-Induced BVP

A 76-year-old man was reported to suffer from increasing imbalance over the past 2.5 months (21). His examination revealed finger-nose-dysmetria, an unsteady gait with a leftward tendency, a positive Romberg test (unable to stand with feet together and arms outstretched while eyes are closed), and increased instability on difficult gait tasks (heel-to-toe-walk). His past medical history included myocardial infarction, paroxysmal atrial fibrillation, peripheral vascular disease, chronic obstructive lung disease, arterial hypertension, and chronic kidney disease. On re-evaluation of his medications, the patient was found to have been inadvertently taking the loading dose of 3 × 400 mg of amiodarone ever since the drug was started 2.5 months ago during a hospitalization. His amiodarone was consequently stopped, and his ataxia slowly improved over a few weeks, with complete resolution after 5 months. The patient had reported that he could walk without difficulty but “felt drunk” without using a cane. Although no vestibular function tests were performed in this patient, the description of his symptoms is consistent with BVP rather than, e.g., cerebellar ataxia, since the latter typically leads to a more severe disturbance of gait and stance and since finger-nose-dysmetria in elderly patients is a rather unspecific finding.

A previously active 95-year-old lady was reported to notice progressive gait instability 8 months after initiating an amiodarone treatment for her paroxysmal atrial fibrillation (22). She was not suitable for treatment with a beta blocker even at small doses because of a symptomatic reduction of blood pressure. She was initiated on amiodarone 200 mg three times a day for 1 week, followed by 200 mg twice a day for 1 week, and then 200 mg once a day. Fourteen days after the loading dose, she reverted to sinus rhythm. Her amiodarone-induced hypothyroidism was treated with 75 µg thyroxine. On examination, she had a wide-based gait, bilateral dysdiadochokinesia, signs of peripheral neuropathy, and “bilateral nystagmus.” The dose of amiodarone was reduced to 100 mg once a day, and 1 month later she felt steadier. One month after the amiodarone was stopped, the gait imbalance had resolved. No explicit tests of vestibular function were performed in this case. The term “bilateral nystagmus” may possibly refer to a bilaterally pathologic head impulse test or may possibly indicate a spontaneous nystagmus oscillating horizontally. The corresponding author of this case report was contacted for further clarification of this “bilateral nystagmus,” but did not respond. Overall, the description of this case is reminiscent of BVP.

A prospective study on neurological toxicity of amiodarone was conducted in New Zealand and included data from 408 patients at the time of its market introduction (10). The authors provided the prescribing doctors with a questionnaire to be filled out at the next patient visit, which included questions about paresthesia, neuropathy, tremor, vertigo, ataxia, impaired intellect, muscle weakness, cerebrovascular accident, transient ischemic attack, diplopia, speech disorder, migraine, and other neurological problems. Within this cohort, 28% of the patients had at least one neurological adverse effect. Of note, the most frequent adverse effects were vertigo (9%) and gait imbalance (9%). Furthermore, these two symptoms were highly significantly correlated. Vertigo and gait imbalance were also the most frequent causes of withdrawal or reduction of amiodarone in this patient cohort. The gait imbalance was described as an unsteadiness, and five patients were reported having falls. Three patients were reported as “cerebellar ataxia.” However, since vestibular function tests or other differentiating tests such as the Romberg test were not reported, there is little evidence to locate the lesion to the cerebellum in these cases.

Among a patient cohort with relatively high daily maintenance dose (15), ataxia/gait instability was reported in about 7% of the patients. In one patient, the authors noted the presence of nystagmus, but did not further describe the nature of this nystagmus nor did they perform any vestibular tests. Therefore, similar to other studies, in hindsight it may well be suspected that the reported symptoms of ataxia/gait instability were actually (at least partially) caused by BVP.

Another study (9) that analyzed medical records with a retrospective design described 11 patients who were referred to the neurology clinic after the start of an amiodarone treatment and had a plausible amiodarone-related averse effect. These patients were identified among a total of 707 patients receiving amiodarone within one county. Of those, two patients had gait ataxia/instability. However, among nine further patients with possible amiodarone-induced toxicity but who were not referred to the neurology clinic, five suffered from gait instability. Including those cases, the authors calculated an overall prevalence of amiodarone neurotoxicity of 2.8%. Comparing the two groups with and without amiodarone neurotoxicity, there was no difference in age, sex, type of arrhythmia, and daily dose. However, the length of time receiving therapy was a significant risk factor for amiodarone toxicity, in accordance with a previous meta-analysis that found that exposure to amiodarone therapy for at least 12 months doubled the odds of neurologic adverse effects with placebo (despite low doses of 150–330 mg/day) (16).

Further support for the adverse effect of amiodarone on gait stability comes from a recent study which examined the risk of falls in patients diagnosed with atrial fibrillation (23). The authors examined patients aged above 60 years with a history of atrial fibrillation and subdivided them into two groups: those with no history of falls in the previous year and those with a history of one or more falls in the previous year. Among the clinical and epidemiological parameters assessed with multivariate logistic regression, the use of amiodarone could be identified as an independent risk factor for falls.

## Direct Evidence for Amiodarone-Induced BVP

In 2017, the first case of amiodarone-induced BVP was published (4). A 73-year-old man presented to the neurotology clinic with progressive gait imbalance, beginning 6 months after starting amiodarone therapy. Previous clinical investigations including repeated MR imaging excluded alternative pathologies such as cerebellar/brainstem infarction or atrophy and only yielded peripheral neuropathy as diagnosis. Vestibular function testing revealed a bilaterally pathologic head impulse test, profoundly reduced responses on caloric videonystagmography and severely reduced vestibular ocular reflex gain as well as pathological compensatory saccades bilaterally on video head impulse testing. After amiodarone discontinuation, in contrast to a previous case of suspected amiodarone-induced BVP described above, his symptoms only partially resolved. A possible explanation for this may be the fact that he had been taken amiodarone for more than 3 years. A single-center retrospective evaluation of 14 patients treated with amiodarone who were referred to a vertigo center found that 6 of these patients (43%) had BVP (4), which is a surprisingly high prevalence. A very recent retrospective multicenter study in five dizziness clinics (24) approached the subject of amiodarone-induced BVP from a different perspective: The authors analyzed 126 patients with “idiopathic” BVP (i.e., of previously unknown etiology) and found that 15 of these patients were actually taking amiodarone. In all of these patients, the gait instability was progressive over time and two of them reported repeated falls. This prevalence of amiodarone intake of 12% within a cohort of patients with so-called “idiopathic” BVP lies far above an expected random coincidence, and further supports the data of a previous report from a single center.

## Possible Mechanisms of Amiodarone Neuro-/Ototoxicity

There are no histopathologic reports of the effects of amiodarone on the vestibular nerve or the labyrinth. It is therefore not yet possible to pinpoint the exact location of the lesion in amiodarone-induced BVP along the vestibular system pathways.

Histologic studies of sural nerve biopsies reported both axonal degeneration and demyelination (25). A histopathological report on two patients with amiodarone-induced neuropathy (25) reported loss of myelinated fibers, the presence of lysosomal inclusion bodies in Schwann cells, and widening of Ranvier nodal gaps. Schwann cell abnormalities seemed to precede the breakdown of myelin, suggesting that amiodarone-induced neuropathy could be described as a schwannopathy. These changes are likely a result of the effects of amiodarone upon the lysosomal system. They correspond to the observation in animal studies that amiodarone has strong inhibitory effects on lysosomal phospholipases A1 and A2 responsible for catabolizing phospholipids (26), causing formation of the characteristic lysosomal bodies. Further manifestations of this interference of amiodarone with the lysosomal system are microdepositions of lipofuscin in the cornea and the skin frequently encountered in amiodarone-treated patients (Table 1) as well as hepatic and pulmonary toxicity (26).

Experimental animal studies with amiodarone (27) indicate that, in common with other amphiphilic drugs, its distribution among tissues is restricted by vascular barriers, such as the blood–brain barrier, whereas its pathologic cellular effects can be found with a dose-related intensity in regions located outside these barriers, such as dorsal root, area postrema, myenteric plexus, and gasserian and autonomic ganglia. This would be in accordance to the predilection of the amiodarone neurotoxicity for the peripheral nervous system *vs*. the central nervous system, since the blood–nerve barrier is less tightly controlled than the blood–brain barrier (25). Clinical or subclinical disease causing blood–nerve permeability changes may also underly this observation, since a clinical study of amiodarone-associated neuropathy reported two cases with a history of diabetes mellitus (28).

In summary, older reports on amiodarone-induced symptoms of vertigo and gait instability lack a description of vestibular function test results. Recent evidence from retrospective studies including vestibular function testing in patients taking amiodarone could identify the drug as the hitherto unsuspected potential cause of a relatively large proportion of cases with “idiopathic” BVP. Therefore, patients who receive amiodarone should be monitored with vestibular function testing in order to recognize potential adverse effects on the vestibular system and allow an informed decision on a possible drug reduction or withdrawal. Furthermore, in order to precisely determine the dynamics and the prevalence of amiodarone-induced BVP, a prospective study of vestibular function in patients taking amiodarone is recommended.

## Author Contributions

RG conceived, drafted, and revised the manuscript.

## Conflict of Interest Statement

The author declares that the research was conducted in the absence of any commercial or financial relationships that could be construed as a potential conflict of interest.

## Footnotes

^1^NIH, National Library of Medicine. Available from: https://livertox.nlm.nih.gov/Amiodarone.htm

## References

[1] Gurkov R Jerin C Flatz W Maxwell R Superior canal dehiscence syndrome: diagnosis with vestibular evoked myogenic potentials and fremitus nystagmus. HNO (2018) 66(Suppl 1):28–33. 10.1007/s00106-017-0441-x 29242950

[2] Guinand N Boselie F Guyot JP Kingma H. Quality of life of patients with bilateral vestibulopathy. Ann Otol Rhinol Laryngol (2012) 121:471–7. 10.1177/000348941212100708 22844867

[3] van de Berg R van Tilburg M Kingma H. Bilateral vestibular hypofunction: challenges in establishing the diagnosis in adults. ORL J Otorhinolaryngol Relat Spec (2015) 77:197–218. 10.1159/000433549 26366566

[4] Ruehl RM Guerkov R Amiodarone-induced gait unsteadiness is revealed to be bilateral vestibulopathy. Eur J Neurol (2017) 24:e7–8. 10.1111/ene.13203 28102050

[5] Kashima A Funahashi M Fukumoto K Komamura K Kamakura S Kitakaze M Pharmacokinetic characteristics of amiodarone in long-term oral therapy in Japanese population. Biol Pharm Bull (2005) 28:1934–8. 10.1248/bpb.28.1934 16204949

[6] Pollak PT Wee V Al-Hazmi A Martin J Zarnke KB. The use of amiodarone for in-hospital cardiac arrest at two tertiary care centres. Can J Cardiol (2006) 22:199–202. 10.1016/S0828-282X(06)70896-0 16520848 PMC2528918

[7] Allen LaPointe NM Dai D Thomas L Piccini JP Peterson ED Al-Khatib SM Antiarrhythmic drug use in patients <65 years with atrial fibrillation and without structural heart disease. Am J Cardiol (2015) 115:316–22. 10.1016/j.amjcard.2014.11.005 25491240 PMC4293335

[8] Epstein AE Olshansky B Naccarelli GV Kennedy JI Jr Murphy EJ Goldschlager N Practical management guide for clinicians who treat patients with amiodarone. Am J Med (2015) 129(5):468–75. 10.1016/j.amjmed.2015.08.039 26497904

[9] Orr CF Ahlskog JE. Frequency, characteristics, and risk factors for amiodarone neurotoxicity. Arch Neurol (2009) 66:865–9. 10.1001/archneurol.2009.96 19597088

[10] Coulter DM Edwards IR Savage RL. Survey of neurological problems with amiodarone in the New Zealand Intensive Medicines Monitoring Programme. N Z Med J (1990) 103:98–100. 2314744

[11] Anderson NE Lynch NM O’Brien KP. Disabling neurological complications of amiodarone. Aust N Z J Med (1985) 15:300–4. 10.1111/j.1445-5994.1985.tb04040.x 3864421

[12] Greene HL Graham EL Werner JA Sears GK Gross BW Gorham JP Toxic and therapeutic effects of amiodarone in the treatment of cardiac arrhythmias. J Am Coll Cardiol (1983) 2:1114–28. 10.1016/S0735-1097(83)80338-6 6685151

[13] Morady F Sauve MJ Malone P Shen EN Schwartz AB Bhandari A Long-term efficacy and toxicity of high-dose amiodarone therapy for ventricular tachycardia or ventricular fibrillation. Am J Cardiol (1983) 52:975–9. 10.1016/0002-9149(83)90515-5 6637851

[14] Charness ME Morady F Scheinman MM. Frequent neurologic toxicity associated with amiodarone therapy. Neurology (1984) 34:669–71. 10.1212/WNL.34.5.669 6538658

[15] Palakurthy PR Iyer V Meckler RJ. Unusual neurotoxicity associated with amiodarone therapy. Arch Intern Med (1987) 147:881–4. 10.1001/archinte.1987.00370050077013 3034178

[16] Vorperian VR Havighurst TC Miller S January CT. Adverse effects of low dose amiodarone: a meta-analysis. J Am Coll Cardiol (1997) 30:791–8. 10.1016/S0735-1097(97)00220-9 9283542

[17] Cairns JA Connolly SJ Roberts R Gent M. Randomised trial of outcome after myocardial infarction in patients with frequent or repetitive ventricular premature depolarisations: CAMIAT. Canadian Amiodarone Myocardial Infarction Arrhythmia Trial Investigators. Lancet (1997) 349:675–82. 10.1016/S0140-6736(96)08171-8 9078198

[18] Ahmed S Rienstra M Crijns HJ Links TP Wiesfeld AC Hillege HL Continuous vs episodic prophylactic treatment with amiodarone for the prevention of atrial fibrillation: a randomized trial. JAMA (2008) 300:1784–92. 10.1001/jama.300.15.1784 18854540

[19] Julian DG Camm AJ Frangin G Janse MJ Munoz A Schwartz PJ Randomised trial of effect of amiodarone on mortality in patients with left-ventricular dysfunction after recent myocardial infarction: EMIAT. European Myocardial Infarct Amiodarone Trial Investigators. Lancet (1997) 349:667–74. 10.1016/S0140-6736(96)09145-3 9078197

[20] Trip MD Wiersinga W Plomp TA. Incidence, predictability, and pathogenesis of amiodarone-induced thyrotoxicosis and hypothyroidism. Am J Med (1991) 91:507–11. 10.1016/0002-9343(91)90187-3 1951413

[21] Willis MS Lugo AM. Amiodarone-induced neurotoxicity. Am J Health Syst Pharm (2009) 66:567–9. 10.2146/ajhp080196 19265185

[22] Hindle JV Ibrahim A Ramaraj R. Ataxia caused by amiodarone in older people. Age Ageing (2008) 37:347–8. 10.1093/ageing/afn063 18385185

[23] Santos AC Nobre MR Nussbacher A Rodrigues GH Gebara OC Azul JB Predictors of the risk of falls among elderly with chronic atrial fibrillation. Clinics (Sao Paulo) (2012) 67:305–11. 10.6061/clinics/2012(04)02 22522754 PMC3317245

[24] Gurkov R Manzari L Blodow A Wenzel A Pavlovic D Luis L Amiodarone-associated bilateral vestibulopathy. Eur Arch Otorhinolaryngol (2017) 275(3), 823–25. 29282523 10.1007/s00405-017-4858-3

[25] Jacobs JM Costa-Jussa FR. The pathology of amiodarone neurotoxicity. II. Peripheral neuropathy in man. Brain (1985) 108(Pt 3):753–69. 10.1093/brain/108.3.753 2994809

[26] Heath MF Costa-Jussa FR Jacobs JM Jacobson W. The induction of pulmonary phospholipidosis and the inhibition of lysosomal phospholipases by amiodarone. Br J Exp Pathol (1985) 66:391–7. 2992568 PMC2041094

[27] Costa-Jussa FR Jacobs JM. The pathology of amiodarone neurotoxicity. I. Experimental studies with reference to changes in other tissues. Brain (1985) 108(Pt 3):735–52. 2994808 10.1093/brain/108.3.735

[28] Martinez-Arizala A Sobol SM McCarty GE Nichols BR Rakita L. Amiodarone neuropathy. Neurology (1983) 33:643–5. 10.1212/WNL.33.5.643 6302557

